# De novo variations of *ANK1* gene caused hereditary spherocytosis in two Chinese children by affecting pre-mRNA splicing

**DOI:** 10.1186/s12887-022-03795-0

**Published:** 2023-01-16

**Authors:** Yang Wang, Lan Huang, Yao Zhu, Xizhou An, Jiacheng Li, Jiangwei Zhen, Jie Yu

**Affiliations:** 1 Department of Hematology and Oncology, Children’s Hospital of Chongqing Medical University, National Clinical Research Center for Child Health and Disorders, Ministry of Education Key Laboratory of Child Development and Disorders, Children’s Hospital of Chongqing Medical University, 136 Zhong shan er lu, Yu zhong district, Chongqing, 400014 China; 2 Chongqing Key Laboratory of Pediatrics, Chongqing, China; 3 Department of Endocrinology, Shenzhen Samii International Medical Center, Shenzhen, 518000 China

**Keywords:** De novo variation, Hereditary spherocytosis, *ANK1*, Minigene splicing assay

## Abstract

**Background and aims:**

Hereditary spherocytosis (HS) is one of the most common hereditary haemolytic disorders. Here, two unrelated families with the probands displaying typical manifestations of HS were enrolled. Our study aimed to characterize the effect of two novel variants in HS patients on gene splicing to help minimize the rate of misdiagnosis of HS and enhance clinicians’ understanding of the disease.

**Participants and methods:**

A retrospective review was conducted. Peripheral blood samples were collected from all the family members, and genomic DNA was extracted for genetic diagnostics. First, high-throughput sequencing technology was used for the preliminary screening of candidate causative variants. Thereafter, the variants were verified via Sanger sequencing. Furthermore, a pathogenicity analysis of the detected variants was performed including in silico prediction and in vitro experiments. We constructed matched wild-type and mutant-type minigene plasmid of *ANK1* based on HEK293T cells to address the effects of variants on mRNA splicing.

**Results:**

The c.1305 + 2 T > A (family1) and c.1305 + 2del (family2) variants were detected in the *ANK1* gene. These two de novo mutations described by us which have not been reported prior to this study. Moreover, the validation results of splicing reporter systems revealed that the intronic mutations resulted in abnormal pre-mRNA splicing. Specifically, the minigene plasmid expressing the c.1305 + 2 T > A variant transcribed the two aberrant transcripts: r.1305_1306ins1305 + 1_1305 + 229 and r.1305_1306ins1305 + 1_1305 + 552. The minigene plasmid expressing c.1305 + 2del transcribed the two aberrant transcripts: r.1305_1306ins1305 + 1_1305 + 228 and r.1305_1306ins1305 + 1_1305 + 551.

**Conclusion:**

The two de novo variants identified in the *ANK1* gene were the genetic etiology of the probands with HS in our study. Our findings further enrich the HS genotype database and provide a basis for genetic counselling and molecular diagnosis.

**Supplementary Information:**

The online version contains supplementary material available at 10.1186/s12887-022-03795-0.

## Introduction

Hereditary spherocytosis (HS, MIM#612641) is a hereditary genetic disease caused by abnormalities in red blood cell membrane proteins resulting from congenital haemolytic anaemia [1]. HS is mainly characterized by anaemia, jaundice and splenomegaly [2]. Additional features comprise increased erythrocyte osmotic fragility and spherical red blood cell (RBC) count in peripheral blood smears [3]. The symptomatology and clinical outcomes of HS are highly variable. Patients with mild HS might have no clinical manifestations related to anaemia due to the compensatory effect of the bone marrow, causing more erythropoiesis than destruction. Meanwhile, patients with severe HS might suffer from hemolytic crisis or even death [4]. The major genetic mode of HS is autosomal dominant manner, accounting for approximately 75% of cases. In addition, approximately one-fourth of HS patients have no family history of the disease, and the inheritance patterns indicate that HS can be inherited as an autosomal recessive single-gene disorder or as a result of de novo mutations [5]. To date, 5 associated genes associated with HS have been reported: *ANK1*, *SLC4A1*, *SPTA1*, *SPTB* and *EPB42*, encoding ankyrin protein, band-3 protein, alpha-spectrin protein, beta-spectrin protein and erythrocyte membrane protein band 4.2, respectively [6]. In summary, HS shows marked clinical and genetic heterogeneity [7]. The prevalence of HS patients also exhibits a cosmopolitan distribution. Epidemiological studies have shown that the incidence of HS in Europe is approximately 1/2000–1/5000 [8], whereas in China the estimated incidence is 1.39/100000 [9]. Notably, typical symptoms are not present at the same time in most patients with HS, and various factors can easily influence laboratory findings. Thus, diagnosing HS is difficult, and patients are frequently misdiagnosed and underdiagnosed. The incidence of the disease may be much higher than the clinical detection rate. Thus, obtaining a definite diagnosis is challenging, even though HS is not very rare. To the best of our knowledge, unequivocal gene sequencing can be used to confirm the diagnosis of HS [10]. Therefore, the discovery of pathogenic gene variants detected by genetic testing and the detailed and intensive functional analysis associated with these genes might shed further light on HS pathogenesis.

In this article, online prediction tools and minigene constructs were used to further reveal the pathogenicity and characterization of novel variants detected in two Chinese families with HS, more accurately characterizing splicing variants in *ANK1*. This work further explains the relationship between the genotype and phenotype associated with *ANK1* variants in the Chinese population and provides the basis for prenatal diagnostics and genetic counselling.

## Materials and methods

### Subjects

Two families with no genetic relationship were recruited, and both probands presented with progressively deteriorating jaundice at birth or shortly after. Detailed family history was collected for each patient through face-to-face interviews. According to the above data, we constructed genotype-based family pedigrees.

### Genetic testing and analysis

To further clarify the diagnosis, 5 ml blood samples from all family members were collected and processed into EDTA anticoagulation tubes for genomic DNA (gDNA) extraction. A targeted next-generation sequencing panel provided by Shanghai Cinopath Medical Testing Co., Ltd. was applied to capture all exon sequences of ~ 700 genes associated with inherited diseases of the blood and immune system. Briefly, gDNA was extracted using the Blood Gen Midi Kit (CWBIO, Beijing, China) according to the manufacturer’s instructions. The extracted gDNA was next fragmented by sonication with a Qsonica Q800R and amplified to construct next-generation sequencing libraries with a Library Quantification kit (Kapa Biosystems) and an HTP library Preparation kit (Kapa Biosystems). Next, sequencing of DNA fragments was conducted by utilizing a high-throughput sequencer (NovaSeq 6000 Analyser, Illumina, United States). The mean sequencing depth for the targeted sequencing regions was 500-1000×. After passing evaluation by Illumina Sequence Control Software, the raw data were read using Next GENe software (Soft Genetics, Inc., USA). Sequenced FASTQ files were quality-filtered through Trimmomatic (version 0.36). Evaluation of sequence capture effect and genome quality was conducted using Burrows-Wheeler Aligner (BwA, version 0.7.13). Validation of single nucleotide variants and indels (insertions and deletions) of sequences was performed using Free Baves software (version 1.1.0) to obtain both sequencing coverage and accuracy. Subsequently, detected mutations were annotated according to a method reported in the literature [11]. Popular prediction methods were employed to detect the sequence variants in the probands’ samples, as described in a previous study [12]. Confirmatory testing in probands and family members was performed using Sanger sequencing.

### Splicing prediction

Two tools from the online website RDDC RNA Splicer (https://rddc.tsinghua-gd.org/) and SpliceAI (https://spliceailookup.broadinstitute.org/) [13] were used to assess the inferred effect of variants of *ANK1* on splicing.

### Minigene splicing assay

The de novo variants c.1305 + 2 T > A and c.1305 + 2del were obtained by target sequence capture and next-generation sequencing screening. The pMini-CopGFP vector (Hitrobio.tech, Inc., China) was used to construct the mini gene and analyse the effect of the variants on the splicing of the *ANK1* gene. These methods are briefly explained in the following sections. For PCR, 2 μl gDNA was amplified in a 50 μl reaction volume with 1.5 μl of each upstream and downstream primer, 10× reaction buffer 5 μl, KOD-Plus-Neo 1 μl, DNTP 5 μl, MgSO4 3 μl and ddH_2_O 31 μl. The PCR amplification conditions were optimized according to the manufacturer’s instructions. The PCR product was subsequently digested with BamH I and XhoI and cloned into the pMini-CopGFP vector using the same sites. Overnight cultures of the ligated mixture-transformed *E. coli* strain Top10 were grown in LB media at 37 °C, and the transformed bacteria were selected by screening the colonies on kanamycin (100 μg/ml)–containing agar plates. An endotoxin-free plasmid kit (Tian gen, Beijing, China) was employed to prepare endotoxin-free plasmids. The plasmids were verified by Sanger sequencing before subsequent use. All the primer-related information is shown in Supplementary Table S1. PCR conditions are described in Supplementary Materials.

HEK293T cells were cultured in medium supplemented with 10% FBS 100 U/ml penicillin, and 100 U/ml streptomycin at 37 °C in 5% CO2. HEK293T cells in optimal growth conditions were grown to 50–60% confluence in a 6-cm cell culture dish for transduction. Then, the hybrid minigenes were transfected into the HEK293T cells using Lipofectamine® 2000 (Thermo Fisher Scientific, Waltham, MA, CA). The cells were harvested 48 h after transfection, and total RNA was isolated using TRIzol. RT-PCR products were collected and then subjected to Sanger sequencing.

## Results

### Case presentations and follow-up

**Fig. 1:**
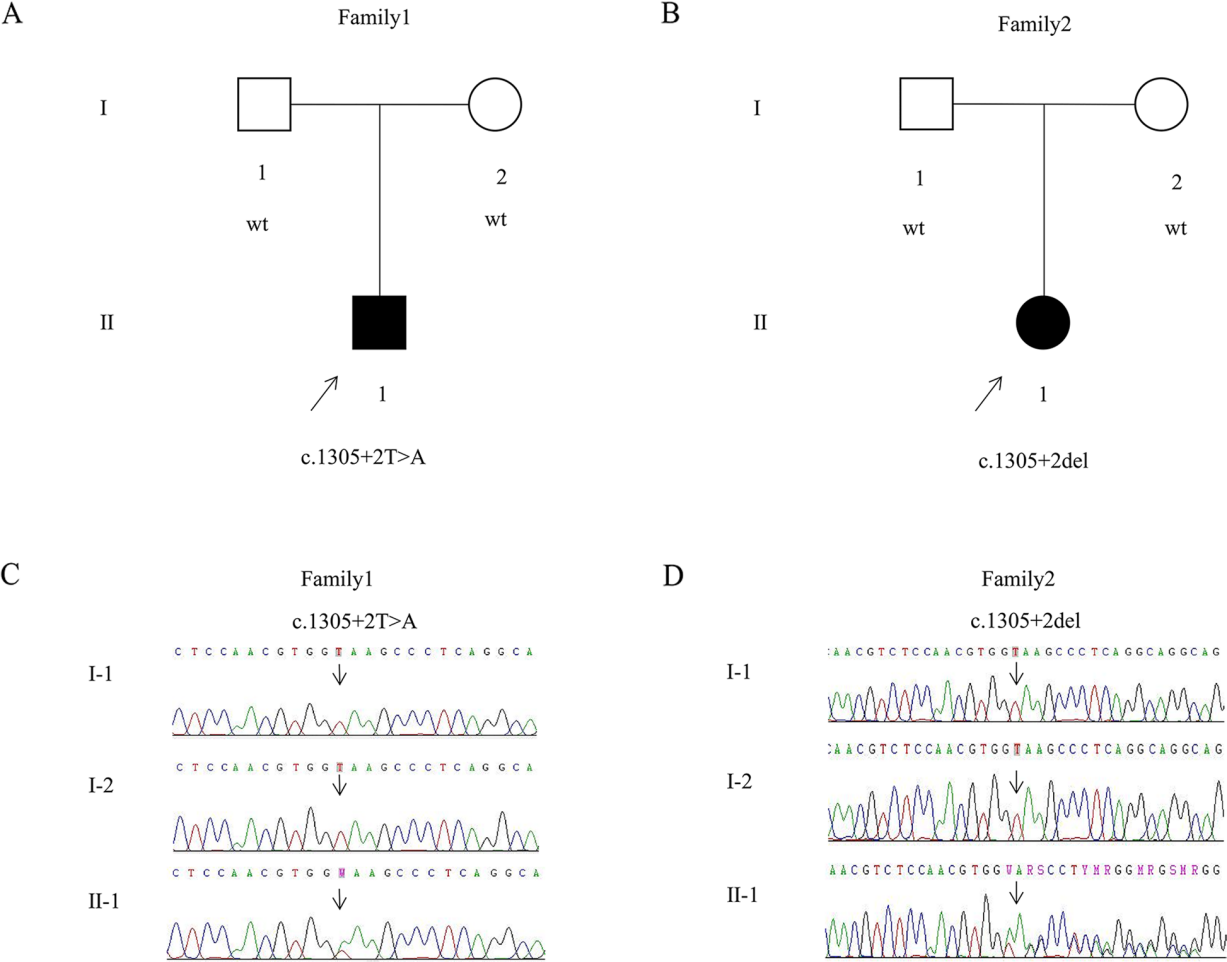
The genetic segregation of variants and the details of *ANK1* mutations in family 1 and family 2. **a**-**b** Pedigrees of proband 1 and proband 2. **c**-**d** The genetic variants carried by both probands are de novo and were not inherited from the patients. Circles represent female family members and squares represent males. Filled symbols designate individuals affected with HS

Patient 1 (II-1, Fig. 1A) was an 8-year-old boy with typical chronic haemolytic anaemia. The patient had experienced episodes of weakness, easy fatigability and skin color abnormalities since birth. Approximately 4 years ago, the child with HS gradually developed splenomegaly, which persists. The proband’s parents were nonconsanguineous and had no history of congenital disorders or recurrent miscarriages. Routine blood tests revealed that the RBC count, haemoglobin concentration and haematocrit level were severely reduced, while erythrocyte distribution width and reticulocyte ratio were increased (Table 1). The blood biochemical examination indicators were not within the normal range (Table 1). Haptoglobin levels were < 0.0583 g/L (normal range, 0.30-2.10 g/L). Blood smears showed mature RBCs of variable size and spherocytes accounted for 2.3%. Direct and indirect antiglobulin test results were negative. In the osmotic fragility tests, compared with the normal controls, the RBCs of the proband displayed a greater osmotic fragility. The morphologic examination of the bone marrow aspirations showed an actively proliferating population of erythroblasts and an increased proportion of nucleated erythrocytes. No abnormalities were found in the blood counts and morphology of blood smears in the patient’s parents based on light microscopy observations. The pediatric patient was closely monitored by regular routine blood analyses and clinical evaluations. During episodes of hemolysis, the patient required RBC transfusion support.

**Table 1:** The clinical and laboratory data of the Chinese patients with HS

Patients	Gender	Age	RBC	Hb	MCV	MCH	MCHC	Ret	RDW	TB
1	M	8y	3.30	89	78.8	27.0	342	0.063	22.1	59.9
2	F	1 m	2.76	77	93.6	28.8	308	0.127	20.3	164.2

Patient 2 (II-1, Fig. 1B) was a 1-month-old girl referred to our hospital because of jaundice after birth that had exacerbated; the patient had demonstrated general listlessness for 1 day. The child’s skin was jaundiced at postnatal day 2. Jaundice may subside after blue light phototherapy and recur after the end of treatment. Furthermore, paleness, mental distress, moderate yellowing of the skin over the body, and hepatosplenomegaly were observed during physical examination on admission. No other remarkable phenotypic presentations were observed. The patient was the parents’ only child. Both parents were 28 years of age, healthy, and had no family medical history. In addition, the routine blood examination and blood biochemical analysis parameters were not within the normal range in patient 2 (Table 1). Direct and indirect antiglobulin test results were negative. Additionally, peripheral blood smears exhibited disparities in RBC size and the presence of spherocytes (approximately 1.5%). No abnormalities were presented in the blood count and morphology analyses of the patient’s parents. Furthermore, the morphologic findings of the bone marrow smear analysis revealed active proliferation with erythroid preponderance with an increase in intermediate and late erythrocytes, mature erythrocytes of varying sizes, and the presence of polychromatic and spherical erythrocytes. After admission, the patient underwent a 0.5 U suspended RBC transfusion to correct the anaemia. After treatment, haemoglobin levels increased to normal, the haemolysis was controlled, the bilirubin levels were restored to normal, and the patient was discharged. The patient is monitored regularly, and aggressive symptomatic treatments are applied during haemolytic episodes.

RBC (normal range, 3.50-5.30 × 10^12/L); Hb, (normal range, 120-158 g/L); MCV (normal range, 77.0-92.0 fL); MCH (normal range, 26.0-33.0 pg); MCHC (normal range, 311-357 g/L); Ret (normal range, 0.5-1.5%); RDW (normal range, < 15.0%); TB (normal range, 0-20.5 μM).

### Molecular and genetic analysis of the pathogenic variants

**Fig. 2:**
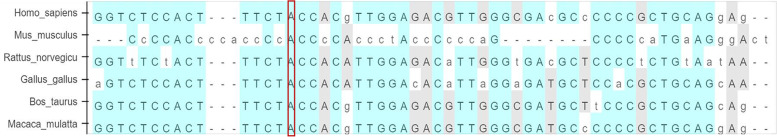
Multiple sequence alignment in the c.1305 + 2 site variant of A*NK1* from different species, including *Homo sapiens*, *Mus musculus*, *Rattus norvegicus*, *Gallus*, *Bos taurus* and *Macaca mulatta*, revealed a high degree of evolutionary conservation. Highly conserved amino acids are shown in the red boxes

Novel variants c.1305 + 2 T > A and c.1305 + 2del (NM_020475.3) in the *ANK1* gene were identified by target sequence capture combined with high-throughput sequencing technology in the probands. Then, Sanger sequencing results indicated that only the two probands carried the splicing variants among the two genealogically unrelated pedigrees, while the other members did not carry the variants, demonstrating that c.1305 + 2 T > A and c.1305 + 2del are de novo variants. Further details are shown in Fig. 1C-D. Collectively, c.1305 + 2 T > A and c.1305 + 2del variants were not detected in the 1000 database, dbsNP database, or EsP exon database. Both variants were classified as pathogenic according to ACMG guidelines (10.1038/gim.2015.30). Most remarkably, the site is highly conserved across different species (Fig. 2).

**Fig. 3:**
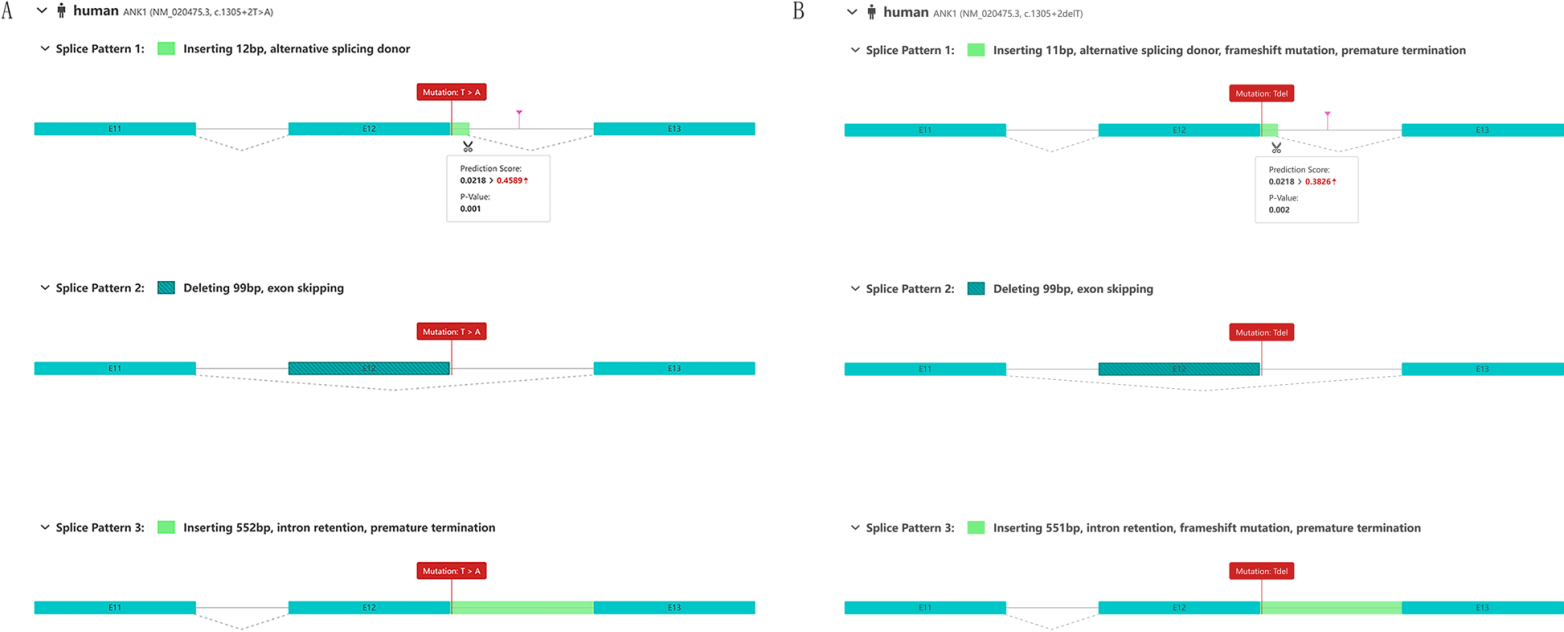
RDDC RNA Splicer prediction results. **a** The c.1305 + 2del variant can produce three splice forms, including an 11 bp insertion, providing an alternative splicing donor, a 99 bp deletion, resulting in exon skipping, and a 551 bp insertion, leading to a premature termination codon. **b** The c.1305 + 2 T > A variant can produce three splice forms, including a 12 bp insertion, providing an alternative splicing donor, a 99 bp deletion, resulting in exon skipping, and a 552 bp insertion, leading to a premature termination codon

Subsequent in-depth analyses were conducted on the two de novo splicing variants. Specifically, the freely available online bioinformatics tools RDDC RNA Splicer and Splice AI were used for in silico analysis to predict the influence of the c.1305 + 2 T > A and c.1305 + 2del variants on *ANK1* splice sites on mRNA splicing. The prediction results of RDDC RNA Splicer are shown here. The c.1305 + 2del variant can produce three splice forms, including an 11 bp insertion, providing an alternative splicing donor, a 99 bp deletion, resulting in exon skipping, and a 551 bp insertion, leading to a premature termination codon. The c.1305 + 2 T > A variant can produce three splice forms, including a 12 bp insertion, providing an alternative splicing donor, a 99 bp deletion, resulting in exon skipping, and a 552 bp insertion, leading to a premature termination codon. For details of RDDC RNA Splicer results are shown in Fig. 3. Otherwise, Splice AI predicted that the c.1305 + 2 T > A variant could lead to donor gain with a score of 0.53 (at − 10 bp distance) and donor loss with a score of 1 (at 2 bp distance). In addition, for the c.1305 + 2del variant, the predictions were donor loss with a score of 1 (at 3 bp distance) and donor gain with a score of 0.45 (at -9 bp distance). It should be mentioned that 100 bp is the max distance for predictions of Splice AI in our study.

### Splicing analysis of *ANK1* c.1305 + 2 T > A and c.1305 + 2del in the Minigene

**Fig. 4:**
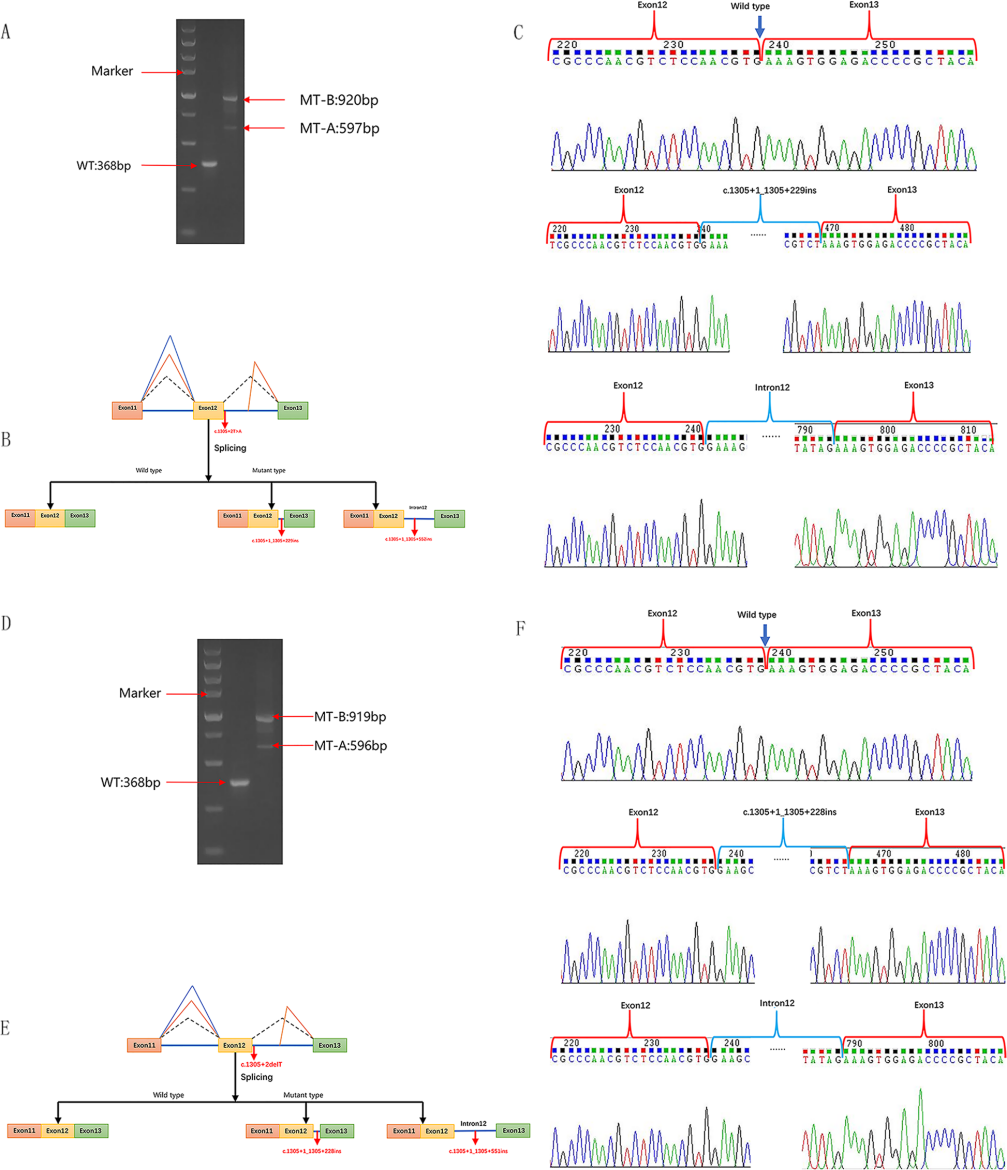
In vitro splicing results from the analysis of potential splice-altering capabilities of the c.1305 + 2 T > A and c.1305 + 2del variants. **a**, **d** Agarose gel electrophoresis of RT–PCR products of wild-type and mutated minigenes of the c.1305 + 2 T > A variant and c.1305 + 2del variant, respectively. The marker represents the DNA ladder. **b**, **e** Schematic diagram of wild-type and mutated minigene fragments. Specifically, the transcriptional mRNA sequence of wild plasmid was consistent with the expectation, including complete exon11, exon12 and exon13. The minigene plasmid expressing the c.1305 + 2 T > A variant transcribed the two aberrant transcripts: r.1305_1306ins1305 + 1_1305 + 229 and r.1305_1306ins1305 + 1_1305 + 552. The minigene plasmid expressing c.1305 + 2del transcribed the two aberrant transcripts: r.1305_1306ins1305 + 1_1305 + 228 and r.1305_1306ins1305 + 1_1305 + 551. **c** Sanger sequencing chromatograms of RT–PCR products of the c.1305 + 2 T > A variant. **f** Sanger sequencing chromatograms of RT–PCR products of the c.1305 + 2del variant

We generated appropriate minigene constructs to explore the effect of variants c.1305 + 2 T > A and c.1305 + 2del on the *ANK1* gene. At 48 h post-transfection, we extracted total RNA from plasmid-transfected 293 T cells according to the manufacturer’s protocol. Specifically, the findings of the experiments were similar but not entirely in agreement with the results of the in silico prediction. By electrophoresis, the full-length amplification product was 368 bp in 293 T cells transfected with the indicated wild-type plasmids (Fig. 4A, D). Meanwhile, the amplification products were 597 bp and 920 bp in 293 T cells transfected with the c.1305 + 2 T > A mutant plasmid (Fig. 4A). In 293 T cells transfected with the c.1305 + 2del mutant plasmid, the amplification products were 596 bp and 919 bp (Fig. 4D).

The PCR products were excised and subjected to Sanger sequencing in this study. In detail, Sanger sequencing of gel-purified amplified DNA fragments showed that the WT plasmid-transcribed mRNA sequences were as expected, containing complete exons11,12, and13. Moreover, the mutant minigene plasmids c.1305 + 2 T > A and c.1305 + 2del transcribed two aberrant transcripts, r.1305_1306ins1305 + 1_1305 + 229 and r.1305_1306ins1305 + 1_1306-1, respectively (Fig. 4C, F; corresponding mRNA splicing diagram shown in Fig. 4B, E).

## Discussion

We enrolled two unrelated families with probands showing typical HS clinical presentations. The two children, both of whom experienced HS onset shortly after birth, demonstrated clinical features of chronic haemolytic anaemia, jaundice, hepatosplenomegaly, and blood transfusion dependence, consistent with previous reports on HS patients [14]. In both patients, a low percentage of spherical red blood cells was shown in peripheral blood smears. Additionally, the parents of the patients carrying de novo mutations had normal clinical characteristics and haematological test results [15, 16]. Comprehensive clinical and genetic analyses were performed to provide detailed characteristics of the patients’ genotypes and phenotypes. The de novo variants c.1305 + 2 T > A and c.1305 + 2del in the *ANK1* gene were identified via target sequence capture combined with high-throughput sequencing technology. The variants were predicted to have a deleterious effect by bioinformatics tools. Furthermore, in vitro minigene experimental validations demonstrated that these variants affected the splicing process of pre-mRNA.

Previous research has shown that erythrocyte membrane protein deficiency caused by pathogenic mutations in the *ANK1*, *SLC4A1*, *SPTA1*, *SPTB*, and *EPB42* genes is the molecular pathogenesis of HS [17]. More importantly, it was previously reported that nonsense mutations account for the majority of known *ANK1* mutations [18]. In our study, the de novo splice variants c.1305 + 2 T > A and c.1305 + 2del in two unrelated Chinese HS pedigrees were identified by high-throughput sequencing. The sequence is highly conserved among various species. Moreover, the two variants that occur at canonical ±1 or 2 splice sites were identified as de novo variants by pedigree analysis. The clinical presentations of the two *ANK1* mutation carriers did fit the criteria for HS. The two variants are extremely rare in normal populations and were assessed as pathogenic according to the ACMG guidelines. The insertions to the coding sequence are likely to lead to premature stop codons, thus affecting the structure and function of the protein. Mutated mRNAs could be degraded by nonsense-mediated decay (NMD) to protect the integrity of the transcriptome and normal mRNAs to control the quantities of unmutated transcripts [19]. The variants were defined as the key pathogenetic events in our study patients. This is because the two unrelated Chinese children carrying the splice variants demonstrated the classic clinical phenotype of HS. Combined with the clinical phenotype and genetic information, we speculated that these two novel splice variations in the *ANK1* gene, escape from NMD or fail to trigger NMD, might affect the protein structure and function. Certainly, further experimental validation is needed to confirm this. Our results further document and expand the database of *ANK1*-causing mutations. Specifically, most mutation analyses in genetic diseases are performed only at the genomic DNA level, and experimental verification of the effect of mutations on mRNA expression and the pre-mRNA splicing process is rare. Here, we evaluated two variants in *ANK1*, c.1305 + 2 T > A and c.1305 + 2del, by in silico and in vitro minigene splicing assays, respectively. The in vitro minigene data were partially in line with the predicted results, as shown in the results section. Therefore, further studies are essential for confirming the functional relevance of these predictions. In addition, both patients in the different lineages carried c.1305 + 2 variants involving a single-base substitution and deletion, suggesting that this variant may be recurrent. These new perspectives need to be verified in larger sample sizes. Remarkably, splicing variants are the genetic cause of HS in several patients, but intronic regions are not commonly included in genetic testing, even if these variants are found in DNA. Genetic testing should be carried out whenever possible to confirm the diagnosis [20]. Further RNA analysis is also recommended for assessing the pathogenicity of the variants to reduce the misdiagnosis and underdiagnosis of HS.

There are no effective pharmacological therapeutics available for HS. The patients in our study were treated with at least one blood transfusion during acute haemolytic episodes. However, it is known that excessive iron accumulation is a greater potential threat than anaemia, ultimately leading to multiorgan failure and death [21]. For patients with profound haemolysis, splenectomy is currently the most effective treatment [22]. Research has revealed that partial/subtotal splenectomy or therapeutic spleen embolization tends to be applied in individualized treatment for children younger than 6 years [23]. Regrettably, some HS patients require regular blood transfusion or are transfusion dependent after splenectomy [24]. Moreover, splenectomy can lead to an increased risk of fatal bacterial infections and venous thrombosis [25]. After decades of intense research, gene therapy is beginning to show promise for treating a wide range of monogenic diseases, especially monogenic haematopoietic disorders [26]. methods are currently the most prevailing and efficient tools in haematopoietic stem cell gene therapy. CRISPR/Cas9 technology can directly repair mutated β-globin protein genes and restore normal expression of β-globin [27, 28]. Furthermore, a previous study (Sara Fañanas-Baquero et al., 2021) found that the CRISPR–Cas9 system and donor recombinant adeno-associated vector delivery reconstituted human haematopoiesis in primary and secondary immunodeficient mice, effectively treating pyruvate kinase deficiency [29]. With advances in modern medicine and personalized therapy, gene therapy must be modified to optimize safety and efficacy profiles. Gene therapy is a promising method for permanently curing currently untreatable diseases [30]. Therefore, identifying novel mutations is essential for understanding genotype–phenotype relationships comprehensively and designing targeted gene therapies in the future.

In conclusion, it is recommended that genetic testing should be performed as early as possible. Specifically, variants in intronic regions deserve more attention. To reduce the incidence of HS, prenatal diagnostics and genetic counselling are necessary for families with affected members. We believe that a cure for HS will be achieved with the continuous optimization of gene therapy.

## Supplementary Information

**Additional file 1.**

**Additional file 2.**

**Additional file 3.**

**Additional file 4.**

## Data Availability

The data analyzed in the current study are available in the GenBank database. The Gene ID of *ANK1* during the current study is 286. The cDNA sequence numbers are NM_020475 and the gDNA sequence numbers are NG_012820.2.

## Abbreviations

HS: Hereditary spherocytosis
RBC: Red blood cell
gDNA: Genomic DNA
WT: Wild type
MT: Mutant type
PCR: Polymerase chain reaction
RT-PCR: Reverse Transcription-Polymerase Chain Reaction
CRISPR: Clustered regularly interspaced short palindromic repeat
M: Male
F: Female
Hb: Hemoglobin
MCV: Mean corpuscular volume
MCH: Mean corpuscular hemoglobin
MCHC: Mean corpuscular hemoglobin concentration
Ret: Reticulocyte count
RDW: Red cell distribution width
TB: Total bilirubin
ACMG: American College of Medical Genetics and Genomics
NMD: Nonsense-mediated decay
