# Supplementary material for: De novo variations of ANK1 gene caused hereditary spherocytosis in two Chinese children by affecting pre-mRNA splicing
Source: BMC Pediatr. 2023 Jan 16;23:23. doi: 10.1186/s12887-022-03795-0 (PMC9841706; doi:10.1186/s12887-022-03795-0)
Supplement: Supplementary file 1 — Additional file 1. [file 12887_2022_3795_MOESM1_ESM.docx]

**Supplementary Table S1. Primer sequences used to amplify *ANK1* genomic fragments**

| **Process** | **Name of Prime** | **Primer sequence (5’→3’)** |
| --- | --- | --- |
| Amplify the pMini- ANK1- WT | ANK1-F | AAGCTTGGTACCGAGCTCGGATCCAATGGCTTTACCCCCTTACACATCGCCT |
|  | ANK1-R | TTAAACGGGCCCTCTAGACTCGAGCTTGGCCTTGGCATTGACTTTGGCTTTG |
| Amplify the pMini- ANK1-1305+2delT mutant | ANK1-1305+2delT -F | AACGTCTCCAACGTGGAAGCCCTCAGGCAGGCAG |
|  | ANK1-1305+2delT -R | TTCCACGTTGGAGACGTTGGGCGACGCCCCCCG |
| Amplify the pMini- ANK1-1305+2T>A mutant | ANK1-1305+2T>A -F | AACGTGGaAAGCCCTCAGGCAGGCAGGGGCTT |
|  | ANK1-1305+2T>A -R | TGAGGGCTTtCCACGTTGGAGACGTTGGGCGA |
