# Supplementary figures and images for: De novo variations of ANK1 gene caused hereditary spherocytosis in two Chinese children by affecting pre-mRNA splicing
Source: BMC Pediatr. 2023 Jan 16;23:23. doi: 10.1186/s12887-022-03795-0 (PMC9841706; doi:10.1186/s12887-022-03795-0)

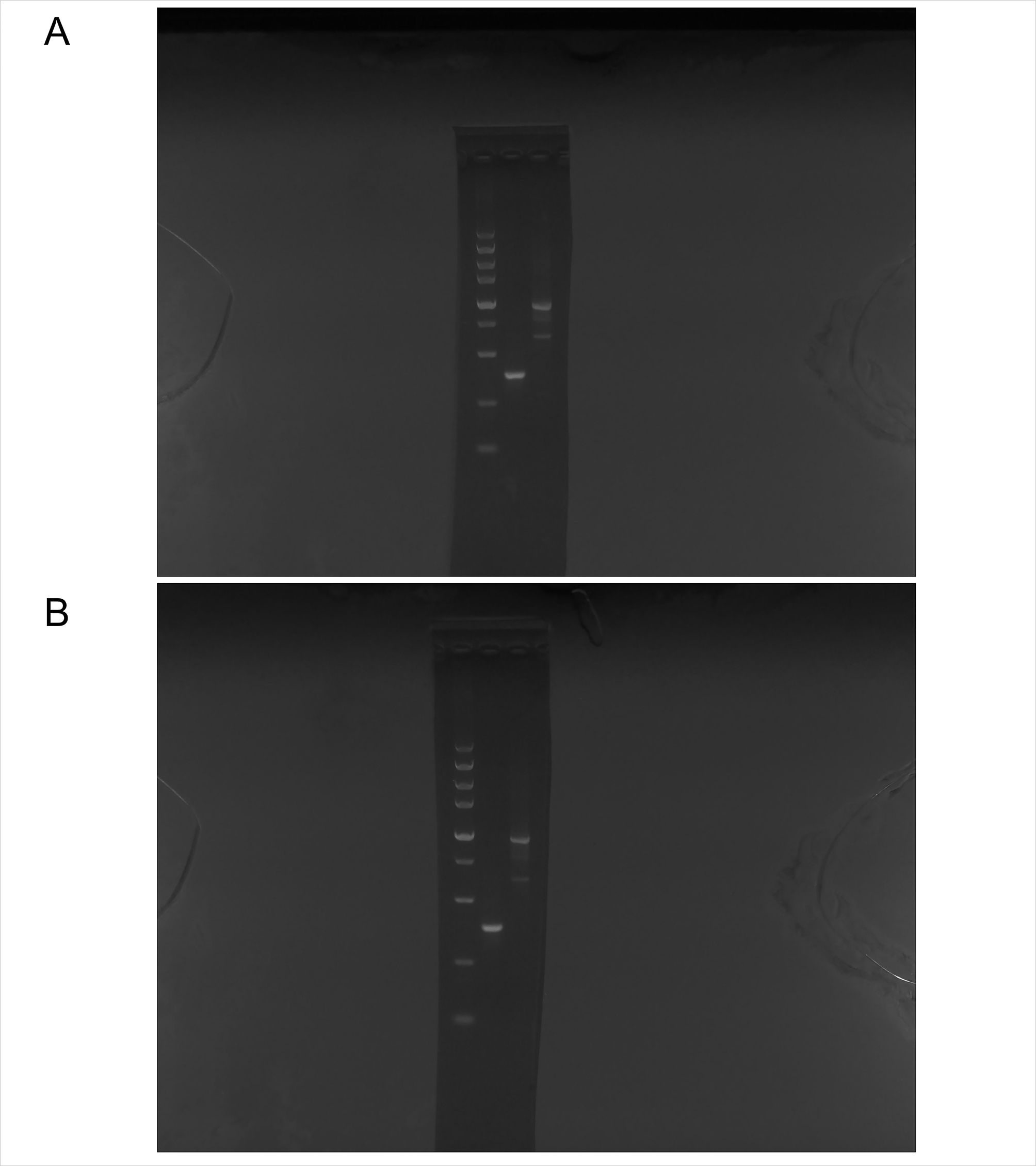

Supplement: Supplementary file 2 — Additional file 2. [file 12887_2022_3795_MOESM2_ESM.tif]
