# Supplementary material for: De novo variations of ANK1 gene caused hereditary spherocytosis in two Chinese children by affecting pre-mRNA splicing
Source: BMC Pediatr. 2023 Jan 16;23:23. doi: 10.1186/s12887-022-03795-0 (PMC9841706; doi:10.1186/s12887-022-03795-0)
Supplement: Supplementary file 3 — Additional file 3. [file 12887_2022_3795_MOESM3_ESM.docx]

**Supplementary material 2.**

**Title: Original graphs of PCR product on agarose gels**

**Legend:**

**A)original graphs of PCR product of NM_020475：c.1305+2T>A and WT in *ANK1* B)original graphs of PCR product of NM_020475：c.1305+2del and WT in *ANK1***

The original images are presented below:


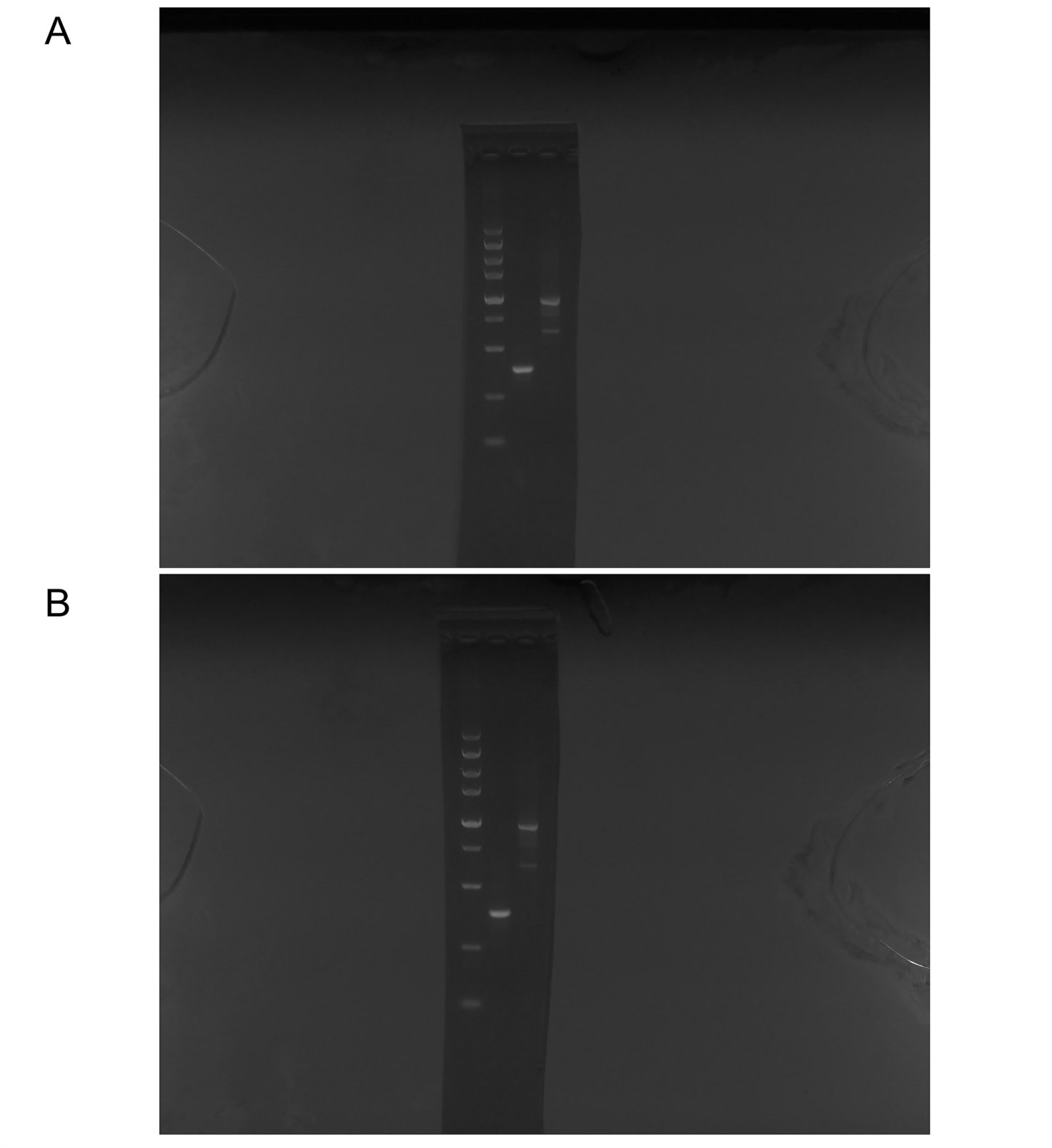


**Supplementary Figure 1.** **Original graphs of PCR product on agarose gels**

A)original graphs of PCR product of NM_020475：c.1305+2T>A and WT in *ANK1* B)original graphs of PCR product of NM_020475：c.1305+2del and WT in *ANK1*
