# Supplementary material for: De novo variations of ANK1 gene caused hereditary spherocytosis in two Chinese children by affecting pre-mRNA splicing
Source: BMC Pediatr. 2023 Jan 16;23:23. doi: 10.1186/s12887-022-03795-0 (PMC9841706; doi:10.1186/s12887-022-03795-0)
Supplement: Supplementary file 4 — Additional file 4. [file 12887_2022_3795_MOESM4_ESM.doc]

**Supplementary material 1.**

Detailed methods and results of the *in vitro* minigene experimental validation.

**1.Diagram of the backbone of pMini-CopGF expression plasmid.**


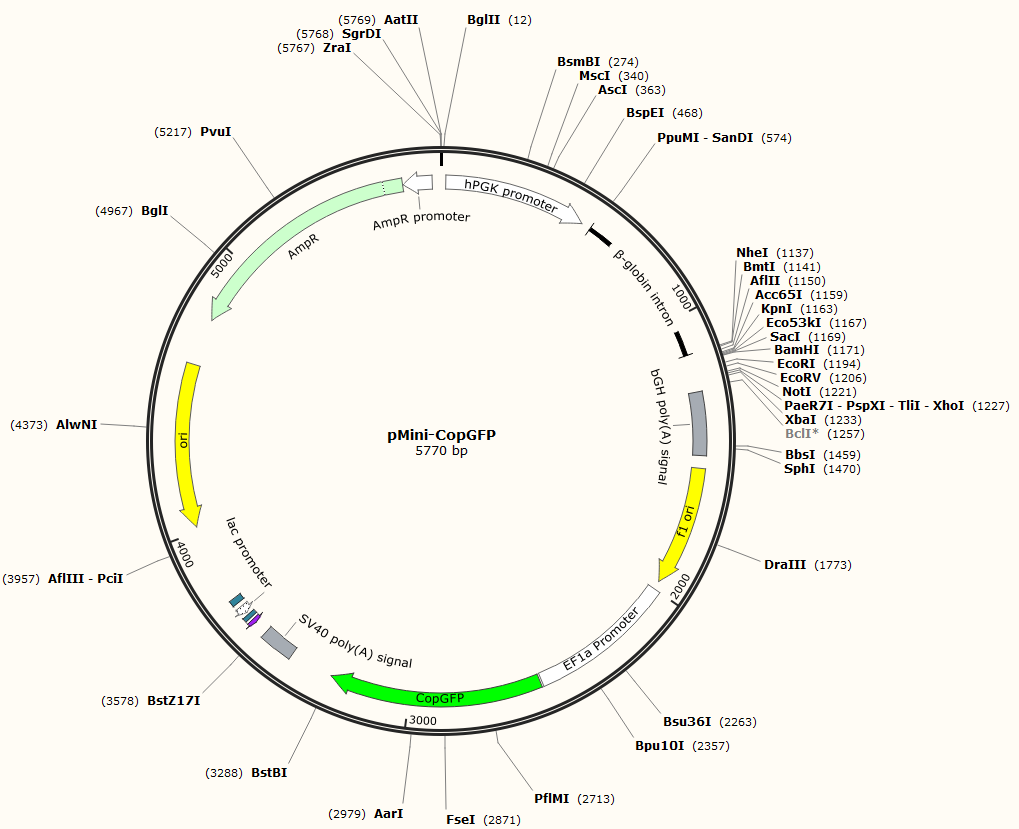


**2.Plasmid Vector Construction.**

Vector: pMini-CopGFP

Restriction enzyme cutting sites: BamHI/XhoI

**3.Inserted sequences**

**3.1.ANK1-WT** aatggctttacccccttacacatcgcctgcaaaaagaaccacgtccgtgtcatggagctgctgctgaagacgggagcctcgatcgacgcggtcaccgaggtaggagagactgcagaggagcctgggggcaggcctgtggtctcccccgacccaccttctccaagagtcgctctttgtagctttgtatggctgcaggggtgtgtgtgtctgcaagtctggactgctggaaggaatgaatcaggggtgactaagacaagggaacatgagtcaagtctcatggagccttccaagatcagacacaatttttgcaagttgtaagagtgggcacgacttgcccacagtaatttttttctccaaccattttcttggaagcaaacccacctcatatagctcccttttcttggaaaatcatttgtgaaagcgttagatactgtcaggttctctcttcttcaggggagcggaaagactcccacttatcggactctgcctttgtctgtttcagtctggcctgacacctctccacgtggcctccttcatggggcaccttcccatcgtgaagaacctcctgcagcggggggcgtcgcccaacgtctccaacgtggtaagccctcaggcaggcaggggcttgctcccctagaccaagagctcagagggcagctttcaccagtgctcccagtcctactctctggggacagagctacgattcccagaacagccaatgcttcagactgggggaggacatccctgtgtactgacgctttctggattaaatttctcacacatccgtaaaccttggaggctttgttttggacaataattttggaacgtctgtaagtgttcactgggaatacatttacatttttgaatattcattagctctggggtttacttttaagtgtacctagaatacacagacacacacataaactcatatacacgcatgcacctgcacgtatgtgcacacacacacatacacacatacatgctcatgcacacatgcacacataccaggctaaagagctgcctgctggggcaactccacctcttggacaaaaacccaccaagccactccagactgcacttcctagctctgtgtgtgccgttttggacagcaggcatgtatgaacagttctataattttgttattttatagaaagtggagaccccgctacacatggcagccagagccgggcacacggaagtggccaaatatttactccagaacaaagccaaagtcaatgccaaggccaag

**3.2.ANK1-MT(NM_020475：c.1305+2T>A)**

aatggctttacccccttacacatcgcctgcaaaaagaaccacgtccgtgtcatggagctgctgctgaagacgggagcctcgatcgacgcggtcaccgaggtaggagagactgcagaggagcctgggggcaggcctgtggtctcccccgacccaccttctccaagagtcgctctttgtagctttgtatggctgcaggggtgtgtgtgtctgcaagtctggactgctggaaggaatgaatcaggggtgactaagacaagggaacatgagtcaagtctcatggagccttccaagatcagacacaatttttgcaagttgtaagagtgggcacgacttgcccacagtaatttttttctccaaccattttcttggaagcaaacccacctcatatagctcccttttcttggaaaatcatttgtgaaagcgttagatactgtcaggttctctcttcttcaggggagcggaaagactcccacttatcggactctgcctttgtctgtttcagtctggcctgacacctctccacgtggcctccttcatggggcaccttcccatcgtgaagaacctcctgcagcggggggcgtcgcccaacgtctccaacgtggAaagccctcaggcaggcaggggcttgctcccctagaccaagagctcagagggcagctttcaccagtgctcccagtcctactctctggggacagagctacgattcccagaacagccaatgcttcagactgggggaggacatccctgtgtactgacgctttctggattaaatttctcacacatccgtaaaccttggaggctttgttttggacaataattttggaacgtctgtaagtgttcactgggaatacatttacatttttgaatattcattagctctggggtttacttttaagtgtacctagaatacacagacacacacataaactcatatacacgcatgcacctgcacgtatgtgcacacacacacatacacacatacatgctcatgcacacatgcacacataccaggctaaagagctgcctgctggggcaactccacctcttggacaaaaacccaccaagccactccagactgcacttcctagctctgtgtgtgccgttttggacagcaggcatgtatgaacagttctataattttgttattttatagaaagtggagaccccgctacacatggcagccagagccgggcacacggaagtggccaaatatttactccagaacaaagccaaagtcaatgccaaggccaag

**3.3. ANK1-MT(NM_020475：c.1305+2del)**

aatggctttacccccttacacatcgcctgcaaaaagaaccacgtccgtgtcatggagctgctgctgaagacgggagcctcgatcgacgcggtcaccgaggtaggagagactgcagaggagcctgggggcaggcctgtggtctcccccgacccaccttctccaagagtcgctctttgtagctttgtatggctgcaggggtgtgtgtgtctgcaagtctggactgctggaaggaatgaatcaggggtgactaagacaagggaacatgagtcaagtctcatggagccttccaagatcagacacaatttttgcaagttgtaagagtgggcacgacttgcccacagtaatttttttctccaaccattttcttggaagcaaacccacctcatatagctcccttttcttggaaaatcatttgtgaaagcgttagatactgtcaggttctctcttcttcaggggagcggaaagactcccacttatcggactctgcctttgtctgtttcagtctggcctgacacctctccacgtggcctccttcatggggcaccttcccatcgtgaagaacctcctgcagcggggggcgtcgcccaacgtctccaacgtggaagccctcaggcaggcaggggcttgctcccctagaccaagagctcagagggcagctttcaccagtgctcccagtcctactctctggggacagagctacgattcccagaacagccaatgcttcagactgggggaggacatccctgtgtactgacgctttctggattaaatttctcacacatccgtaaaccttggaggctttgttttggacaataattttggaacgtctgtaagtgttcactgggaatacatttacatttttgaatattcattagctctggggtttacttttaagtgtacctagaatacacagacacacacataaactcatatacacgcatgcacctgcacgtatgtgcacacacacacatacacacatacatgctcatgcacacatgcacacataccaggctaaagagctgcctgctggggcaactccacctcttggacaaaaacccaccaagccactccagactgcacttcctagctctgtgtgtgccgttttggacagcaggcatgtatgaacagttctataattttgttattttatagaaagtggagaccccgctacacatggcagccagagccgggcacacggaagtggccaaatatttactccagaacaaagccaaagtcaatgccaaggccaag

**4. Normal transcriptional result sequence as expected**

PCR amplification of normal group expected sequence of 368bp: (upper-case text = vector transcript sequence; lower-case text = target gene exon transcript sequence)

GGCTAACTAGAGAACCCACTGCTTACTGGCTGCTAGCGTTTAAACTTAAGCTTGGTACCGAGCTCGGATCC aatggctttacccccttacacatcgcctgcaaaaagaaccacgtccgtgtcatggagctgctgctgaagacgggagcctcgatcgacgcggtcaccgagtctggcctgacacctctccacgtggcctccttcatggggcaccttcccatcgtgaagaacctcctgcagcggggggcgtcgcccaacgtctccaacgtgaaagtggagaccccgctacacatggcagccagagccgggcacacggaagtggccaaatatttactccagaacaaagccaaagtcaatgccaaggccaag

**5. Sanger sequencing of the RT-PCR products of WT:**

AATGGCTTTACCCCCTTACACATCGCCTGCAAAAAGAACCACGTCCGTGTCATGGAGCTGCTGCTGAAGACGGGAGCCTCGATCGACGCGGTCACCGAGTCTGGCCTGACACCTCTCCACGTGGCCTCCTTCATGGGGCACCTTCCCATCGTGAAGAACCTCCTGCAGCGGGGGGCGTCGCCCAACGTCTCCAACGTGAAAGTGGAGACCCCGCTACACATGGCAGCCAGAGCCGGGCACACGGAAGTGGCCAAATATTTACTCCAGAACAAAGCCAAAGTCAATGCCAAGGCCAAG

**6. Sanger sequencing of the RT-PCR products of c.1305+2T>A variant：r.1305_1306ins1305+1_1305+229:**

AATGGCTTTACCCCCTTACACATCGCCTGCAAAAAGAACCACGTCCGTGTCATGGAGCTGCTGCTGAAGACGGGAGCCTCGATCGACGCGGTCACCGAGTCTGGCCTGACACCTCTCCACGTGGCCTCCTTCATGGGGCACCTTCCCATCGTGAAGAACCTCCTGCAGCGGGGGGCGTCGCCCAACGTCTCCAACGTGGAAAGCCCTCAGGCAGGCAGGGGCTTGCTCCCCTAGACCAAGAGCTCAGAGGGCAGCTTTCACCAGTGCTCCCAGTCCTACTCTCTGGGGACAGAGCTACGATTCCCAGAACAGCCAATGCTTCAGACTGGGGGAGGACATCCCTGTGTACTGACGCTTTCTGGATTAAATTTCTCACACATCCGTAAACCTTGGAGGCTTTGTTTTGGACAATAATTTTGGAACGTCTAAAGTGGAGACCCCGCTACACATGGCAGCCAGAGCCGGGCACACGGAAGTGGCCAAATATTTACTCCAGAACAAAGCCAAAGTCAATGCCAAGGCCAAG

**7. Sanger sequencing of the RT-PCR products of c.1305+2T>A variant：r.1305_1306ins1305+1_1305+552：**

AATGGCTTTACCCCCTTACACATCGCCTGCAAAAAGAACCACGTCCGTGTCATGGAGCTGCTGCTGAAGACGGGAGCCTCGATCGACGCGGTCACCGAGTCTGGCCTGACACCTCTCCACGTGGCCTCCTTCATGGGGCACCTTCCCATCGTGAAGAACCTCCTGCAGCGGGGGGCGTCGCCCAACGTCTCCAACGTGGAAAGCCCTCAGGCAGGCAGGGGCTTGCTCCCCTAGACCAAGAGCTCAGAGGGCAGCTTTCACCAGTGCTCCCAGTCCTACTCTCTGGGGACAGAGCTACGATTCCCAGAACAGCCAATGCTTCAGACTGGGGGAGGACATCCCTGTGTACTGACGCTTTCTGGATTAAATTTCTCACACATCCGTAAACCTTGGAGGCTTTGTTTTGGACAATAATTTTGGAACGTCTGTAAGTGTTCACTGGGAATACATTTACATTTTTGAATATTCATTAGCTCTGGGGTTTACTTTTAAGTGTACCTAGAATACACAGACACACACATAAACTCATATACACGCATGCACCTGCACGTATGTGCACACACACACATACACACATACATGCTCATGCACACATGCACACATACCAGGCTAAAGAGCTGCCTGCTGGGGCAACTCCACCTCTTGGACAAAAACCCACCAAGCCACTCCAGACTGCACTTCCTAGCTCTGTGTGTGCCGTTTTGGACAGCAGGCATGTATGAACAGTTCTATAATTTTGTTATTTTATAGAAAGTGGAGACCCCGCTACACATGGCAGCCAGAGCCGGGCACACGGAAGTGGCCAAATATTTACTCCAGAACAAAGCCAAAGTCAATGCCAAGGCCAAG

**8. Sanger sequencing of the RT-PCR products of c.1305+2del variant：r.1305_1306ins1305+1_1305+228:**

AATGGCTTTACCCCCTTACACATCGCCTGCAAAAAGAACCACGTCCGTGTCATGGAGCTGCTGCTGAAGACGGGAGCCTCGATCGACGCGGTCACCGAGTCTGGCCTGACACCTCTCCACGTGGCCTCCTTCATGGGGCACCTTCCCATCGTGAAGAACCTCCTGCAGCGGGGGGCGTCGCCCAACGTCTCCAACGTGGAAGCCCTCAGGCAGGCAGGGGCTTGCTCCCCTAGACCAAGAGCTCAGAGGGCAGCTTTCACCAGTGCTCCCAGTCCTACTCTCTGGGGACAGAGCTACGATTCCCAGAACAGCCAATGCTTCAGACTGGGGGAGGACATCCCTGTGTACTGACGCTTTCTGGATTAAATTTCTCACACATCCGTAAACCTTGGAGGCTTTGTTTTGGACAATAATTTTGGAACGTCTAAAGTGGAGACCCCGCTACACATGGCAGCCAGAGCCGGGCACACGGAAGTGGCCAAATATTTACTCCAGAACAAAGCCAAAGTCAATGCCAAGGCCAAG

**9. Sanger sequencing of the RT-PCR products of c.1305+2del variant：r.1305_1306ins1305+1_1305+551：**

AATGGCTTTACCCCCTTACACATCGCCTGCAAAAAGAACCACGTCCGTGTCATGGAGCTGCTGCTGAAGACGGGAGCCTCGATCGACGCGGTCACCGAGTCTGGCCTGACACCTCTCCACGTGGCCTCCTTCATGGGGCACCTTCCCATCGTGAAGAACCTCCTGCAGCGGGGGGCGTCGCCCAACGTCTCCAACGTGGAAGCCCTCAGGCAGGCAGGGGCTTGCTCCCCTAGACCAAGAGCTCAGAGGGCAGCTTTCACCAGTGCTCCCAGTCCTACTCTCTGGGGACAGAGCTACGATTCCCAGAACAGCCAATGCTTCAGACTGGGGGAGGACATCCCTGTGTACTGACGCTTTCTGGATTAAATTTCTCACACATCCGTAAACCTTGGAGGCTTTGTTTTGGACAATAATTTTGGAACGTCTGTAAGTGTTCACTGGGAATACATTTACATTTTTGAATATTCATTAGCTCTGGGGTTTACTTTTAAGTGTACCTAGAATACACAGACACACACATAAACTCATATACACGCATGCACCTGCACGTATGTGCACACACACACATACACACATACATGCTCATGCACACATGCACACATACCAGGCTAAAGAGCTGCCTGCTGGGGCAACTCCACCTCTTGGACAAAAACCCACCAAGCCACTCCAGACTGCACTTCCTAGCTCTGTGTGTGCCGTTTTGGACAGCAGGCATGTATGAACAGTTCTATAATTTTGTTATTTTATAGAAAGTGGAGACCCCGCTACACATGGCAGCCAGAGCCGGGCACACGGAAGTGGCCAAATATTTACTCCAGAACAAAGCCAAAGTCAATGCCAAGGCCAAG

**10. PCR conditions**

The PCR amplification conditions were as follows: a denaturation step at 94°C for 2 min, followed by 35 cycles of denaturing at 98°C for 10s, annealing for 30s at 62°C, extension at 68°C for 1.5 min.
